# The Genomic Patterns of Well-Differentiated Pancreatic Neuroendocrine Tumors (NETs) Identify Sub-Sets for Rational Therapeutic Targeting

**DOI:** 10.3390/ijms27188002

**Published:** 2026-09-08

**Authors:** Ioannis A. Voutsadakis

**Affiliations:** 1 Holden Comprehensive Cancer Center, University of Iowa Hospitals and Clinics, Iowa City, IA 52242, USA; ivoutsadakis@yahoo.com or ivoutsadakis@nosm.ca; 2 Carver College of Medicine, University of Iowa, Iowa City, IA 52242, USA; 3 Section of Internal Medicine, Division of Clinical Sciences, Northern Ontario School of Medicine, Sudbury, ON P3E 2C6, Canada

**Keywords:** neuroendocrine neoplasms, MEN1, DAXX, ATRX, chromosomal instability

## Abstract

The natural history of pancreatic neuroendocrine tumors (NETs) can span decades of slow progression, but it is unpredictable, with a more aggressive course also being common. The two clinical biomarkers currently used to define the grade of a tumor, the Ki67 index and the mitotic index, are not able to fully capture the course of individual patients. This creates an unmet need to discover better prognostic biomarkers to inform therapeutic decisions, as well as therapies with overall survival benefit. Two genomic series of pancreatic NETs were examined to discover sub-sets with divergent characteristics. Primary data were downloaded from the cBioportal cancer genomics portal and analyzed at the individual sample level. Pancreatic NET patients without *MEN1*/*DAXX*/*ATRX* mutations were younger than NET patients with any of these mutations. Pancreatic NETs had consistently low tumor mutation burden but were heterogeneous in their Fraction Genome Altered (FGA), a metric of chromosomal instability (CIN), with one group presenting high FGA and another possessing low to intermediate FGA. FGA did not correlate with Ki67, but high FGA was most prevalent in cases with mutations in *MEN1* and *DAXX* or *ATRX*. *TSC2* mutations were significantly more prevalent in the group with high FGA.

## 1. Introduction

Pancreatic neoplasms comprise two completely different sub-sets, the most common adenocarcinomas and the less common neuroendocrine tumors derived from the endocrine pancreas. While the former are the tenth most prevalent neoplasms in men and the seventh most prevalent in women, with incidence predicted to further increase in the near future with the aging of the population, the latter neoplasms display lower prevalence [1]. Among gastrointestinal tumors, pancreatic neoplasms are second after colorectal carcinomas in incidence [1]. Pancreatic adenocarcinomas represent more than 85% of neoplasms diagnosed in the pancreas, with pancreatic neuroendocrine tumors (NETs) being diagnosed in less than 5% of cases and the rest representing other rare histologies, including adenosquamous carcinomas and undifferentiated carcinomas [2]. The prevalence of neuroendocrine neoplasms has increased many folds in the United States since the inception of the Surveillance Epidemiology and End Results (SEER) database about 50 years ago, reflecting better diagnosis, classification and awareness of these neoplasms [3]. The 5-year survival of pancreatic NETs is 75% in contrast to pancreatic adenocarcinomas, which have a 5-year survival of 11% [4]. Most pancreatic NETs are non-functional, but about 30% produce hormones, such as insulin, glucagon, gastrin, vasoactive intestinal polypeptide (VIP), and somatostatin [5,6,7]. However, functional status is not a consideration in the selection of systemic therapies for metastatic NETs.

Neuroendocrine neoplasms are divided into well-differentiated neuroendocrine tumors (NETs) and the rarer poorly differentiated neuroendocrine carcinomas, which are equivalent to small cell carcinomas of other organs, such as the lung. Besides histologic differences, well-differentiated neoplasms differ from neuroendocrine carcinomas in genomic features, including frequent p53 and RB tumor suppressor loss in carcinomas [8]. Well-differentiated NETs are classified into grade 1, grade 2 and grade 3 based on the number of mitoses and the expression of the proliferation marker Ki67 [9]. The cut-offs of Ki67 are less than 3%, 3% to 20% and more than 20% for grade 1, grade 2 and grade 3, respectively, and for mitoses, the cut-offs are less than 2, 2 to 20 and more than 20 mitoses per 10 high power fields, respectively [9]. Localized grade 2 tumors have a strikingly different recurrence risk dependent on the specific Ki67 level and therefore are further categorized into grade 2a, with Ki67 between 3% and 10%, and grade 2b NETs, with Ki67 between 10% and 20% [10]. Pancreatic NETs are commonly diagnosed at the metastatic stage as primary tumors tend to be asymptomatic. A minority of cases are diagnosed at a localized stage, either incidentally or due to obstruction or other symptoms related to hormone secretion. Despite metastatic dissemination, NETs are characterized in most cases by a long natural history, with slow growth and periods of stability. However, even grade 1 and grade 2 metastatic NETs may possess histologic heterogeneity that is not fully captured by diagnostic biopsies and the limited number of pathology specimens available for histologic, Ki67 and mitotic number evaluations. In addition, in localized pancreatic NETs, size is a significant determinant of recurrence, and grade 2 tumors differ in their recurrence depending on the spectrum of Ki67 (above versus below 10%) [11]. The current study examines genomic features of pancreatic NETs based on published series to discover pathogenic associations with potential therapeutic implications and to supplement existing predictors.

## 2. Results

The multi-institute series of pancreatic well-differentiated neuroendocrine tumors included 98 patients. The mean age in the series was 57.3 years, and about two-thirds of the patients were male (Table 1). The cohort contained mostly (about 80%) localized tumors of grade 1 or grade 2 and mostly (87%) non-functional tumors. The mutational landscape of the series was dominated by mutations in three genes: *MEN1* (36.7%), *DAXX* (22.4%) and *ATRX* (10.2%; Table 2). Two other genes, *PTEN* and *SETD2*, had mutations in more than 5% of the cases, while mutations in the other genes were less common (Table 2). Overall, more than half of the cases (50 of 98 cases, 51%) in this series had mutations in one of the three frequently mutated genes. Mutations in *DAXX* and *ATRX* were mutually exclusive, with no cases showing co-existing mutations, but either could co-exist with mutations in *MEN1*. Two-thirds of *DAXX*-mutated cases also had *MEN1* mutations, and 30% of *ATRX*-mutated cases also had *MEN1* mutations. When categorized according to the presence of *MEN1* mutations (*n* = 18, 18.4% of patients in the series), *DAXX*/*ATRX* mutations (*n* = 14, 14.3% of patients in the series), both (*MEN1* with either *DAXX* or *ATRX* mutations, *n* = 18, 18.4% of patients in the series) or none of the three genes mutated (*n* = 48, 49%), there were a few notable clinical differences in the four groups. The two groups that possessed *MEN1* mutations with or without *DAXX*/*ATRX* mutations were older (mean age 62.6 and 62.3 years, respectively) compared with the groups without *MEN1* mutations (Table 1). The two groups with *DAXX* or *ATRX* mutations, with or without *MEN1* mutations, had higher rates of stage IV disease, extra-pancreatic spread, and vascular and perineural invasion than the two groups without *DAXX* or *ATRX* mutations. These same groups with *DAXX* or *ATRX* mutations possessed the most *PTEN* mutations, the fourth most frequent mutation in the series, compared with the two other groups (Table 2). Despite adverse characteristics such as stage IV disease, extra-pancreatic spread, and vascular and perineural invasion being more frequent in the groups with *DAXX* or *ATRX* mutations (statistically significant for extra-pancreatic spread and vascular invasion), all five cases with grade 3 tumors belonged to the group with no *MEN1* or *DAXX*/*ATRX* mutations (Table 1). Survival outcomes were immature, with only 15 deceased patients in the series, and there were no statistically significant differences between the four groups (χ^2^ test *p* = 0.82). 

**Figure 1:**
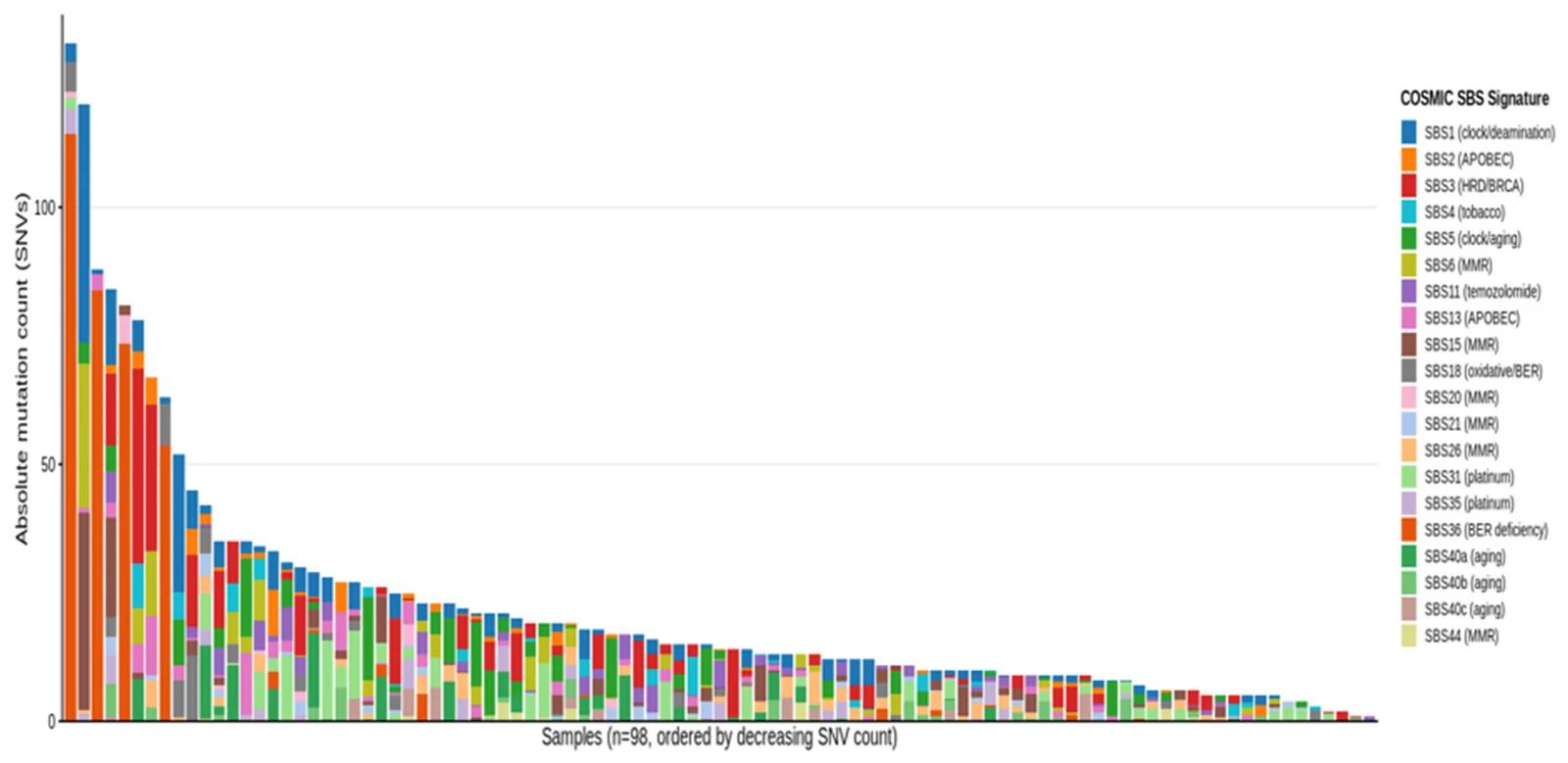
COSMIC SBS signature contribution in the cases of the multi-institute cohort. Cases are ordered according to decreasing number of single nucleotide variants (SNVs). Among the nine cases with the highest number of SNVs on the far left, 4 cases had a significant contribution (>80%) from SBS36 associated with base excision repair (BER) defects.

The mean tumor mutation burden (TMB) of the tumors in the multi-institute series was very low (0.8 mutations/Mb, standard deviation: 0.8). The evaluation of COSMIC SBS signatures in the series disclosed signature SBS36, associated with base excision repair (BER) deficiency (frequently resulting from loss of function *MUTYH* mutations), as the most prevalent signature, contributing 16% of the total single nucleotide variants (SNVs) in the series (Figure 1, Table 3). These were concentrated in 12.4% of the samples, with the most significant portion of the signature observed in four of the nine samples with the highest mutation burden (Table 4). Notably, though, even in these samples, TMB was low (ranging between 1.9 and 4.1), and no *MUTYH* mutations were detected in the cases with high SBS36 contribution or any other cases in the series. All four groups categorized according to the presence of *MEN1* and *DAXX*/*ATRX* mutations were represented in the nine samples with the highest mutation burden. The four samples with the SBS36 signature had a predominance of C>A transversions (81–86%), which are the dominant substitutions of SBS36, while the single base substitutions in the remaining five samples were predominantly C>T transitions (Table 4). Other recurrent mutations in the nine samples included mutations in *TP53*, *PTEN*, and *RB1*, mutations in epigenetic modifiers and mutations in cadherin family (*CDH1*, *CDH6*, *CDH16*, *CDH17*) members (Table 4).

**Figure 2:**
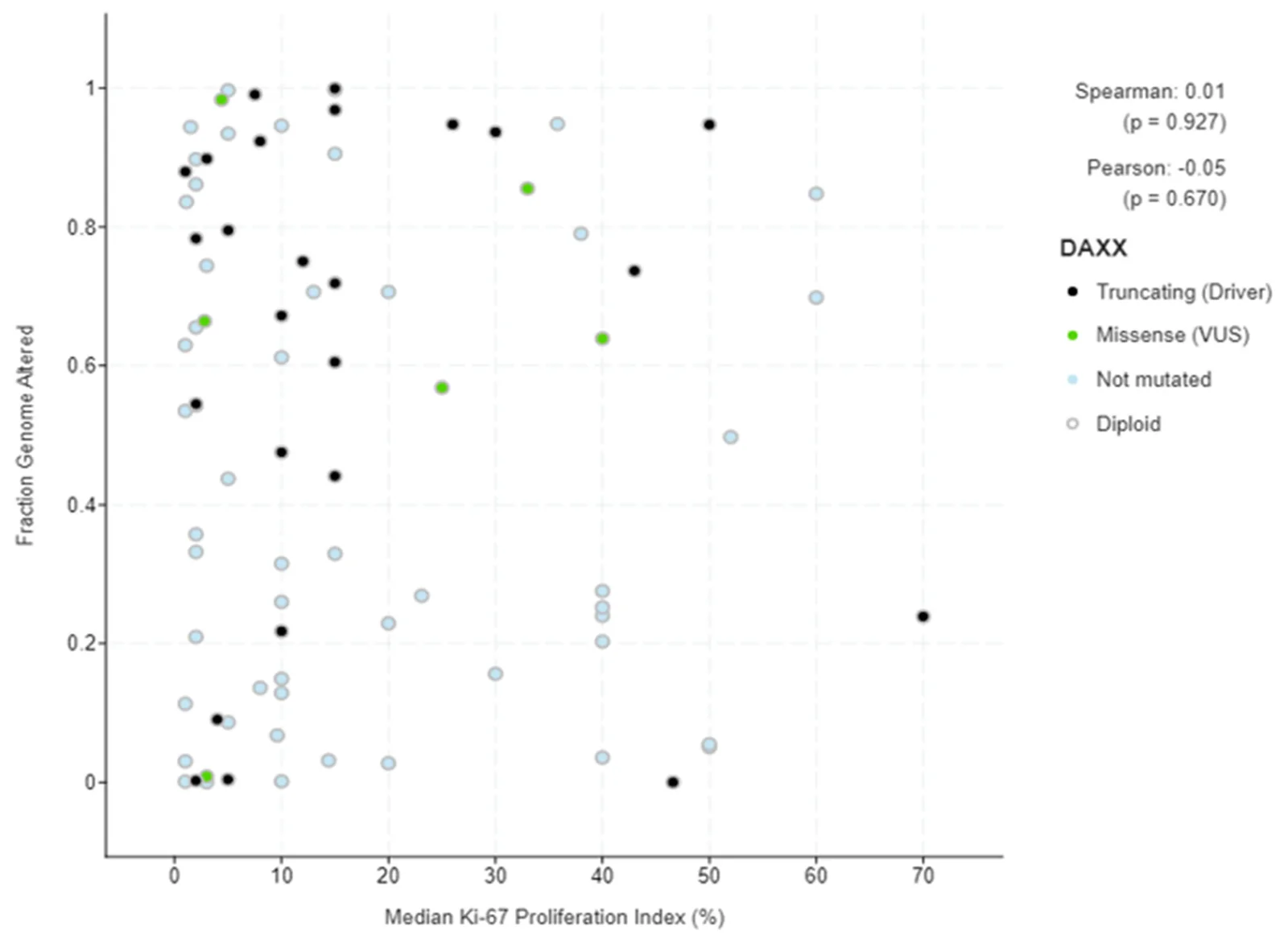
Correlation between Fraction Genome Altered (FGA) and Ki67 in the MSK cohort with DAXX mutations annotated. There is no correlation between FGA and Ki67 (Pearson correlation co-efficient = −0.05, *p* = 0.67).

**Figure 3:**
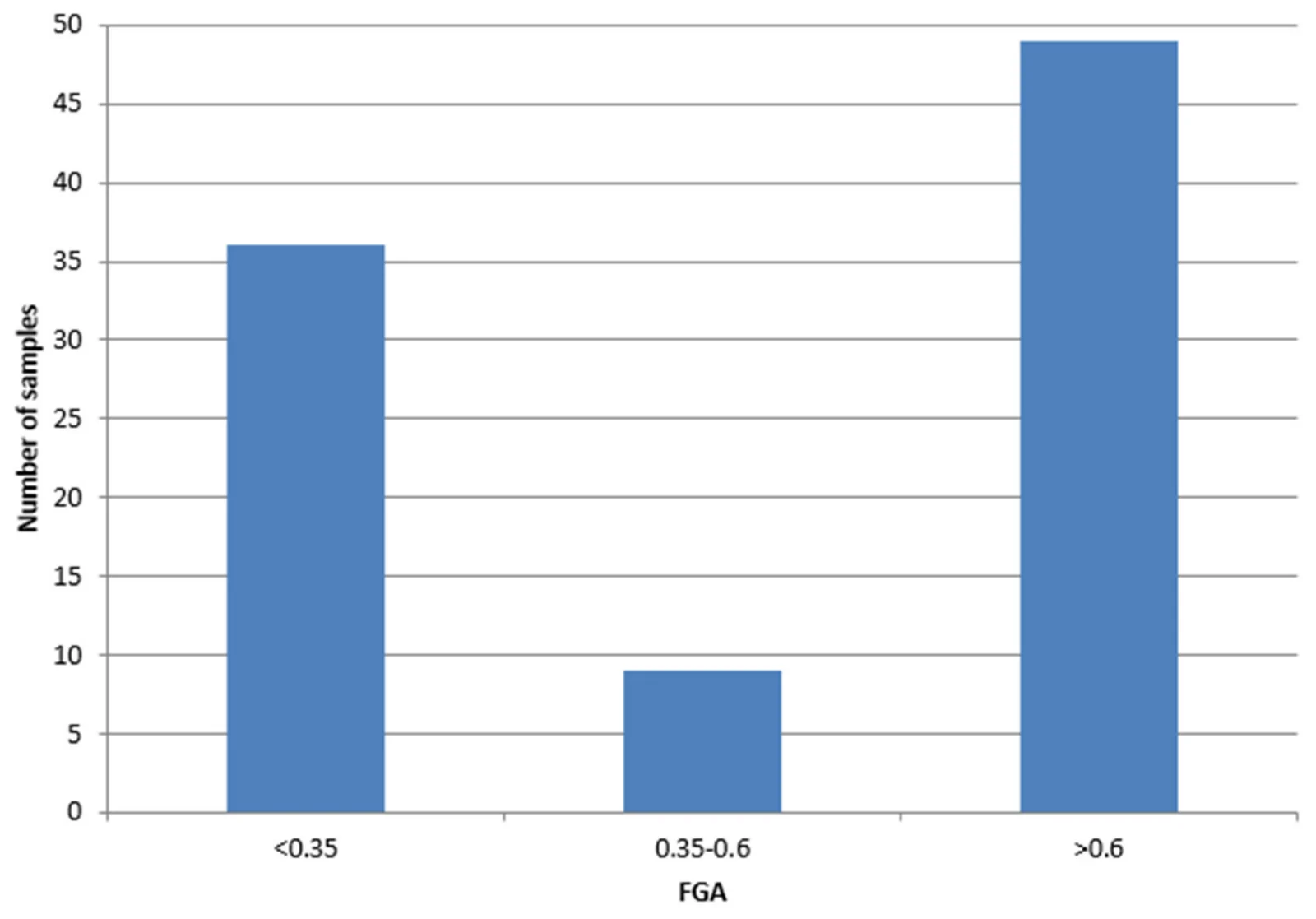
Bimodal distribution of FGA in pancreatic NETs of the MSK cohort. Most cases have FGA below 0.35 or above 0.6. FGA: Fraction Genome Altered.

A second series from the MSKCC (MSK series) included 80 metastatic pancreatic NET patients who contributed 96 samples (some patients contributed two or three samples). The median age was 53.5 years and, in contrast to the multi-institute series, most patients (56.3%) were women (Table 5). Most patients (83.8%) had grade 1 or grade 2 tumors, and most were metastatic to the liver (82.5%) and non-functional (80%). Alterations in *MEN1*, *DAXX* and *ATRX* also dominated the genomic profile of this series. As expected from the targeted panel used in the series, the prevalence of mutations captured was higher than in the multi-institute series that employed whole-exome sequencing. In addition to point mutations, the MSK series examined copy number alterations in the genes of the targeted panel. Alterations in *MEN1* were present in 44 of 80 patients (55%; 41 with mutations and three patients with deep deletions; Table 6 and Table 7). Mutations in *DAXX* were observed in 40% of the patients (32 of 80 patients), and mutations in *ATRX* were observed in 25% of the patients (20 of 80 patients; Table 6). No copy number alterations were observed in either gene, and in this series, five cases had mutations in both *DAXX* and *ATRX*. As expected from the higher prevalence of alterations detected in *MEN1*, *DAXX*, and *ATRX*, when categorized according to the presence of these mutations, the group with both *MEN1* and *DAXX*/*ATRX* alterations contributed 45% of the cases, and the group with neither *MEN1* nor *DAXX*/*ATRX* alterations contained 31.3% of the cases. The two groups with only *MEN1* alterations and only *DAXX* or *ATRX* mutations constituted 10% and 13.8% of the cases in the series, respectively. Similar to the multi-institute series, the patients in the group without *MEN1* or *DAXX*/*ATRX* alterations were younger (Table 5). No other statistically significant differences in clinical characteristics were noted between the four groups. Although there were statistically significant differences in the median mutation count between the groups, with the two groups with *DAXX* or *ATRX* mutations having higher median counts, the mutation rates in all groups were low, consistent with the results from the multi-institute cohort (Table 5). Regarding the median FGA, the group with no *MEN1* or *DAXX*/*ATRX* alterations was an outlier with significantly lower FGA (median 0.2 versus 0.6–0.8 in the other groups, *p* < 0.001). Only three of 30 (10%) in this group had FGA above 0.5, while 34 of 40 samples (85%) in the group with *MEN1* and *DAXX* or *ATRX* alterations had FGA above 0.5 (Fisher’s exact test, *p* < 0.0001). As expected from the fact that the Ki67 index did not differ significantly between the group with no *MEN1* or *DAXX*/*ATRX* alterations and the three other groups, there was no correlation between the Ki67 values in the samples and FGA (Spearman correlation co-efficient = 0.02, *p* = 0.92; Figure 2). The distribution of FGA was U-shaped, with most samples having FGA below 0.35 or above 0.6 (Figure 3). The group of tumors with FGA above 0.6 had statistically significant higher prevalence of mutations in *MEN1* (75% versus 19.4% in tumors with FGA below 0.35, *p* < 0.0001, corrected for multiple comparisons q = 0.002; Table 6), *DAXX* (54.5% versus 19.4% in tumors with FGA below 0.35, *p* = 0.003, which became borderline insignificant after adjustment for multiple comparisons, corrected q = 0.07), and *TSC2* (36.4% versus 3.2% in tumors with FGA below 0.35, *p* = 0.0006, corrected q = 0.01). *PTEN* mutations were numerically more frequent in the group with high FGA but not significant after correction for multiple comparisons (20.5% versus 3.2% in tumors with FGA below 0.35, *p* = 0.03, corrected q = 0.7). In contrast, the group with low FGA below 0.35 had numerically higher mutation rates in *SETD2,* which was insignificant after correction for multiple comparisons (29% versus 9.1% in the group with high FGA, *p* = 0.03, corrected q = 0.7; Table 6). Copy number alterations did not differ significantly between the groups with high and low FGA (Table 7). Besides treatment with somatostatin analogs, which differed between the groups (*p* = 0.04), no differences in other treatments received were observed between the groups categorized according to the presence of *MEN1* or *DAXX*/*ATRX* alterations (Table 8). Similar to the multi-institute cohort, survival evaluation was immature, with most patients (82.5%) alive at the time of study censoring of clinical data and no significant differences between groups (χ^2^ test *p* = 0.27, Table 8). However, the median overall survival was longer in the group with both *MEN1* and *DAXX*/*ATRX* alterations (73.9 months versus 28.6 to 40.5 months in the three other groups; Kruskal–Wallis test, *p* = 0.01).

In the MSK series, additional genes besides *MEN1*, *DAXX* and *ATRX* had mutations in more than 10% of the cases, including *PTEN* (11.3%), *SETD2* (17.5%), *TP53* (12.5%), *ARID1A* (11.3%) and *TSC2* (21.3%). Mutations in the latter were almost exclusively observed in the groups with *DAXX* or *ATRX* mutations, with or without *MEN1* mutations (χ^2^ *p* = 0.006, borderline insignificant after correction for multiple comparisons, q = 0.09; Table 9). The prevalence of mutations in the four other genes (*PTEN*, *SETD2*, *TP53*, and *ARID1A*) was not statistically different between the four groups. A few additional cancer-associated genes with mutations that had not been observed in the multi-institute series had mutations in 5% to 7.5% of the cases in the MSK series (Table 9). Their prevalence did not show statistically significant differences in the four groups, although this may relate to the fact that the absolute number of these mutations in the cohort was small.

The MSK series also examined copy number alterations in the genes of the targeted panel (Table 10). Deletions in *CDKN2A* and *CDKN2B* cyclin-dependent kinase inhibitor genes were observed in 16.3% and 17.5% of the cases, respectively, and were more prevalent in the groups with *DAXX* or *ATRX* mutations, although the difference did not reach statistical significance (χ^2^ *p* = 0.18 and *p* = 0.2 for *CDKN2A* and *CDKN2B*, respectively). *MEN1* deletions were observed in a few cases (3.8%; Table 10).

**Figure 4:**
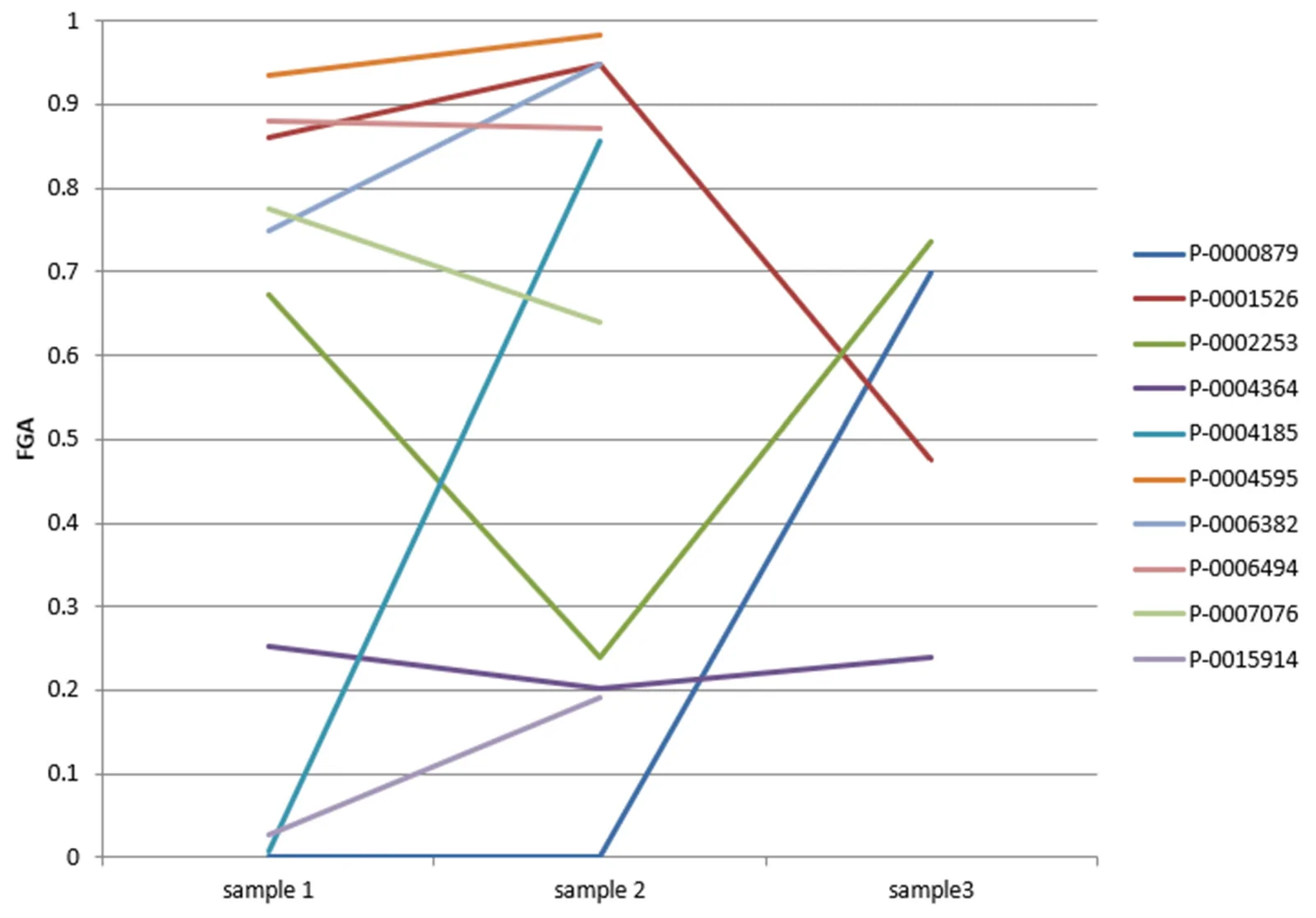
FGA variability in cases with more than one sample in the MSK cohort. FGA: Fraction Genome Altered.

**Figure 5:**
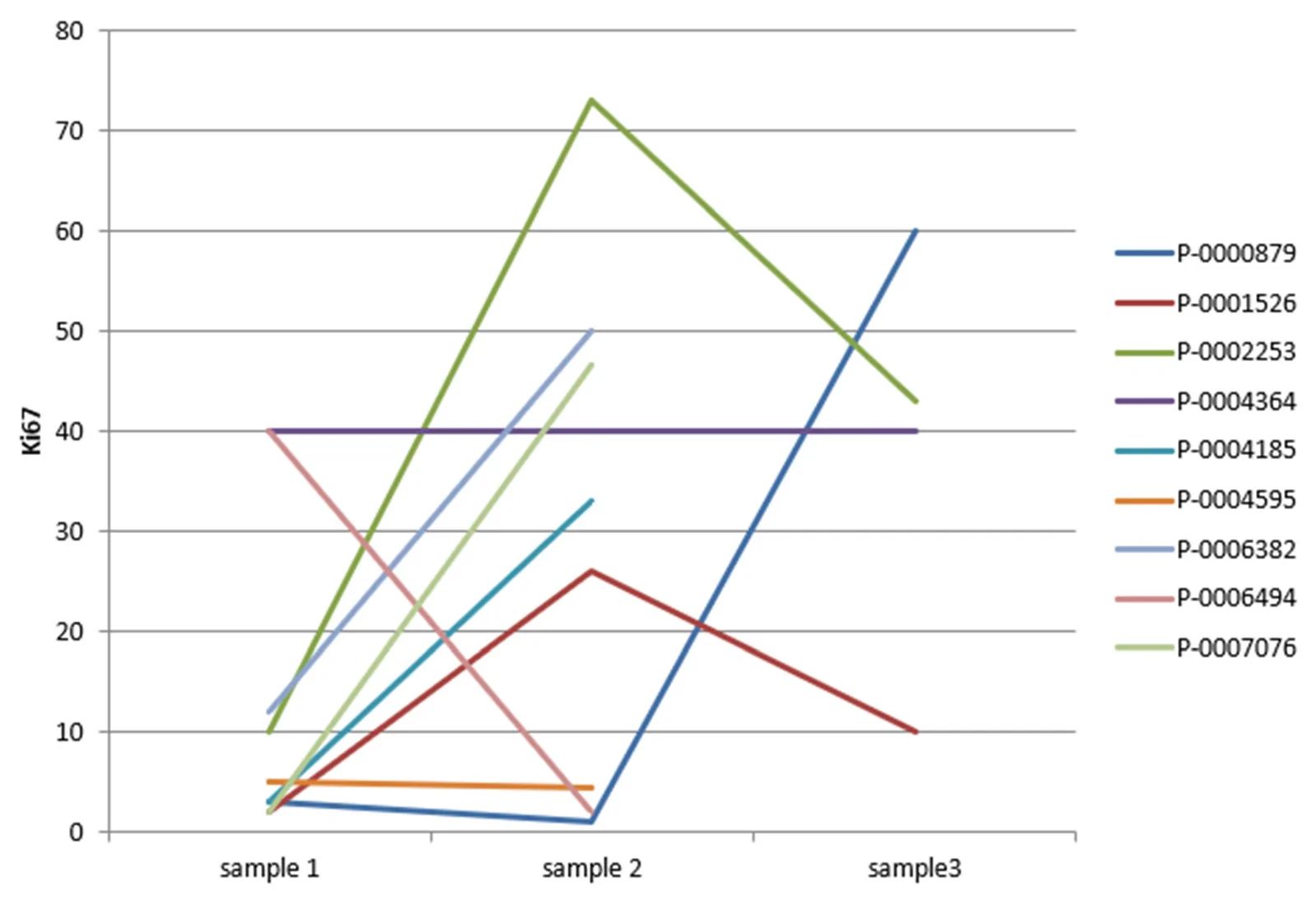
Ki67 variability in cases with more than one sample in the MSK cohort.

Twelve of the 80 patients in the MSK series contributed more than one sample in the study (two samples, *n* = 8 patients, and three samples, *n* = 4 patients). Among the 10 patients with more than one sample and available data for FGA, half of the cases had differences of more than 0.2 between their samples, suggesting topological and temporal heterogeneity in chromosomal instability (Figure 4). Similarly, significant heterogeneity in Ki67 was observed in most cases with more than one sample (Figure 5). 

Acknowledging that the MSK cohort employed a targeted genomic panel, and COSMIC SBS signature evaluation may become skewed due to a small number of mutations in individual samples, an exploratory analysis of COSMIC SBS suggested contributions in sub-sets of cases from signatures related to aging (SBS1, SBS5 and SBS40c), MMR deficiency (SBS6, SBS15, SBS21, SBS26, SBS44), APOBEC deaminase activity (SBS2, SBS13), oxidative damage (SBS18), and platinum drug exposure (SBS31, SBS35). The three samples with the highest mutation burden in the series were dominated by signature SBS11, which is associated with exposure to alkylating agents, such as temozolomide. However, several other patients in the series had confirmed exposure to chemotherapy in their records and did not develop the signature, although the timing of the sampling and specifically whether the biopsy was done before the chemotherapy exposure was not explicitly recorded.

## 3. Discussion

Pancreatic NETs, despite being more indolent than the more common pancreatic adenocarcinomas, are heterogeneous in their biology and prognosis. Pancreatic NETs are almost exclusively well-differentiated tumors. In contrast, poorly differentiated NETs, which have a more uniform, aggressive course, akin to small cell carcinomas of other organs, are rare in the pancreas [12]. Therefore, biomarkers to complement the existing grading system and help in prognosis determination and therapeutic decisions are needed. In the current study, the genomic milieu of pancreatic NET sub-sets classified according to the presence of the most frequent mutations and the level of chromosomal instability, as measured by FGA, was explored by leveraging two publicly available genomic cohorts, with the aim of discovering biomarkers with pathogenic and clinical implications. A notable observation derived from these investigations was that pancreatic NETs without the frequent MEN1, DAXX and ATRX alterations constituted a genomically stable sub-set with low TMB and lower CIN than tumors with these alterations.

The prevalence of *MEN1* and *DAXX*/*ATRX* mutations in a series of 68 pancreatic NETs was 44% and 43%, respectively [13]. In this cohort, similar to the multi-institute cohort examined in the current study, *DAXX* and *ATRX* mutations were mutually exclusive. This suggests that *DAXX* and *ATRX* mutations confer a similar advantage to neoplastic cells, and there is no added benefit of selecting both in the same clones. The presence of *MEN1* and *DAXX* or *ATRX* mutations was associated with better prognosis, which was also suggested by the longer median OS in the MSK series. It is worth mentioning here that formal statistically significant differences for OS are very difficult to demonstrate in any well-differentiated pancreatic NET cohort, even including only metastatic disease patients, given the long natural history of these tumors, with some patients dying with the disease or from complications of treatment, rather than from the disease. mTOR pathway alterations, including mutations in *TSC2*, *PTEN* and *PIK3CA*, were observed in 12 of 68 patients (17.6%; six patients with *TSC2* mutations, five patients with *PTEN* mutations and one patient with a *PIK3CA* mutation). In the two cohorts examined in the current report, *PTEN* and *TSC1*/*TSC2* mutations were also prevalent in a sizeable minority of cases. *PTEN* mutations were observed in 7.1% and 11.3% of cases in the multi-institute and MSK cohort, respectively, and *TSC1*/*TSC2* mutations were present in 4% and 26.3% of cases in the multi-institute and MSK cohort, respectively. In contrast, no *PIK3CA* mutations were observed in either series. The implications of mTOR pathway mutations for the therapeutic targeting of pancreatic NETs with everolimus or other inhibitors of the pathway have not been fully explored. Everolimus is an analog of rapamycin and an inhibitor of the kinase mTOR, which works by inhibiting the kinase when it is part of the mTORC1 complex but has limited inhibitory action against the mTORC2 complex. Pre-clinical studies in cell lines have shown that activation of the pathway due to *PTEN* and *PIK3CA* mutations is associated with mTOR inhibitor sensitivity [14]. A phase 2 clinical trial designed to treat patients with grade 1 and grade 2 NETs of the pancreas and gastrointestinal tract with everolimus or sunitinib according to mutations in the mTOR pathway or in angiogenesis receptors (NCT02315625) was terminated due to poor accrual [15]. A small retrospective cohort of 28 patients with gastroentero-pancreatic NETs treated with everolimus showed a trend towards better Overall Response Rate (ORR) and a statistically significantly better Disease Control Rate (DCR) in a sub-set of patients with alterations in the PI3K/mTOR pathway [16]. Phosphorylated AKT in pancreatic NET samples was also observed in patients who responded to everolimus [17]. In contrast, patients with tumors that stained negative for phosphorylated AKT were resistant to the drug, suggesting that increased pathway activity is a predictive marker of response to everolimus [17]. *TSC2* mutations were more frequent in cases with *DAXX*/*ATRX* mutations, with or without *MEN1* mutations, and in cases with high FGA in the MSK series. Therefore, these could be the groups of pancreatic NET patients who would be expected to respond to everolimus. In estrogen receptor-positive breast cancer, where everolimus is used in combination with hormonal therapy, expression of proteins in the PI3K/mTOR pathway has been associated with response, but no biomarkers have been validated for clinical use [18]. In addition, a low Ki67 index was predictive of response to exemestane/everolimus treatment in this disease [19]. It is worth noting, however, that basket phase 2 trials that selected patients with PI3K/mTOR pathway alterations for treatment with everolimus monotherapy (none with NETs) showed low response rates [20,21]. The first trial of 30 patients with metastatic carcinomas from various primary sites treated with everolimus, who had a median of 2.5 previous lines of treatment, disclosed an ORR of 8% [20]. All the patients in this trial had mutations in *TSC1* (43%), *TSC2* (54%) or *MTOR* (3%). Another trial included 12 metastatic carcinoma patients with a median of three prior treatments and mutations in *STK11* (seven of the 12 patients) or in *NF1* (five of the 12 patients) and showed a complete response in one of the eight evaluable patients (ORR: 12.5%) [21]. Mutations in *TSC1*, *TSC2* and other mTOR pathway components may be good candidates for prediction of treatment efficacy with mTOR inhibitors, but their value needs to be validated prospectively. In contrast to the above series of 68 pancreatic NET patients [12], another study that examined DAXX or ATRX loss by immunohistochemistry (MEN1 not evaluated) in 111 patients with resected pancreatic NETs showed worse relapse-free survival in those with DAXX or ATRX loss [22]. Eighteen patients (16.2%) in this cohort had loss of DAXX or ATRX protein in a mutually exclusive manner, but the mutation status of the two genes was not evaluated [22].

DAXX and ATRX are members of a complex that chaperones variant histone H3.3 to pericentromeric and telomeric chromosome locations [23]. In contrast to carcinomas that protect telomeres through telomerase re-activation, pancreatic neuroendocrine tumors use alternative lengthening of telomeres (ALT) for this purpose, through mutations leading to loss of function of DAXX or ATRX [23]. ALT is also observed in some sarcomas and gliomas [24]. The function of DAXX/ATRX prevents ALT, protecting telomeres by depositing H3.3 through a mechanism aided by recognition of heterochromatin marked by the trimethylation of H3 at the lysine 9 amino acid position (H3K9me3). This recognition is accomplished by the interaction between the ADD (ATRX-DNMT3-DNMT3L) domain of ATRX and the H3K9me3 epigenetic mark. In addition to its role in the prevention of aberrant lengthening of telomeres, the DAXX/ATRX complex has a role in homologous recombination and, therefore, mutations may lead to increased DNA double-strand breaks and CIN [25]. This is relevant in pancreatic NETs with *DAXX*/*ATRX* mutations, which display increased FGA, as shown here.

A study that included both grade 2 and grade 3 well-differentiated NETs and neuroendocrine carcinomas of the gastrointestinal tract and pancreas showed that p53 mutations, as evaluated by immunohistochemistry staining patterns, were associated with poorly differentiated carcinomas and lower survival, while ATRX loss, also evaluated by immunohistochemistry in this study, was not associated with grade or survival outcomes [26]. *TP53* mutations were rare in well-differentiated pancreatic NETs both in the multi-institute cohort of the current report (three of 98 patients, two of them with grade 3 disease and one patient with grade 2 disease) and in another series (3%) [13]. The MSK cohort showed a higher rate of *TP53* mutations (12.5%; five patients with grade 3 disease, five patients with grade 2 disease and two patients with grade 1 disease), suggesting that targeted panels may allow for identification of a higher proportion of these alterations. TP53 mutations, together with RB1 mutations, are more frequent in poorly differentiated neuroendocrine carcinomas, and their presence may imply transformation of well-differentiated NETs.

The distribution of FGA in pancreatic NETs suggests the presence of two sub-sets of tumors, one with low TMB and intermediate or low CIN (genomically stable) and the other with low TMB and high CIN. The latter group had higher rates of mutations in *MEN1*, *DAXX*, *PTEN*, and *TSC2* and numerically higher rates of mutations in *ATRX* and *TP53*, although not statistically significant. As discussed above, DAXX and ATRX participate in DNA damage repair by homologous recombination, and their loss of function promotes double breaks and CIN. Menin, the product of *MEN1*, is also involved in chromosome integrity both as a regulator of alternative splicing and as a repressor of human telomerase reverse transcriptase (hTERT) [27,28]. Menin function in lung cancer cells was required for correct alternative splicing by slowing elongation of nascent mRNA and facilitating interaction with splicing factors [27]. The interaction of mRNA with splicing factors decreases the probability of R-loop formation, which constitutes a risk for DNA damage and genomic instability. The suppression of hTERT by menin is mediated through its recruitment to the promoter of hTERT by transcription factor Sp1 [28]. Degradation of menin by ubiquitin ligase CRL4 in colorectal cancer cells induced the expression of hTERT [28]. Moreover, menin functions during mitosis by regulating correct chromosome cytokinesis [29]. p53 is a key player in the preservation of chromosome stability, and many tumors with *TP53* mutations have high CIN [30]. Loss of function of p53 is critical in the setting of telomere erosion, when a functional p53 would lead to cellular senescence [31]. The sub-set of pancreatic NETs with mutations in any of the three genes, *MEN1*, *DAXX* or *ATRX*, had a distinct genomic signature consistent with alpha islet cells, characterized by up-regulation of transcription factor ARX and promoter hypermethylation and down-regulation of transcription factor PDX1 [32]. ARX is developmentally related to neural tissues, and PDX1 promotes beta cell development in the pancreas, suggesting a mechanistic pathway for the presence of an alpha cell signature in NETs with these mutations. However, despite the genomic signature resembling alpha cells, NETs with *MEN1*, *DAXX* or *ATRX* mutations did not display increased expression of the *GCG* gene, encoding for glucagon [32]. Further clustering inside the three-gene mutant group did not arise depending on the presence of the specific mutations (*MEN1* or *DAXX*/*ATRX* or both), consistent with the binary observation of CIN in the current study. The key role of CIN in pancreatic NETs has been recapitulated in studies that showed specific chromosome loss associations with prognosis [33,34]. One group of pancreatic NETs with isolated loss of chromosome 11 had a better prognosis, while another group with multiple recurrent losses of chromosomes 1, 2, 3, 6, 10, 11, 16 and 22 had an aggressive course [33].

The only cancer-associated gene with higher rates of mutations in the genomically stable group was *SETD2*, which encodes a methyltransferase that performs the trimethylation of histone 3 at lysine 36 (H3K36me3) [35,36]. H3K36 trimethylation is performed in actively transcribed genes, as it requires an interaction between SETD2 through its SRI domain and the phosphorylated carboxyterminal domain of RNA polymerase II [37]. This places SETD2 in a pathway that intersects the scaffolding function of menin during mRNA elongation; therefore, mutations of the two proteins may affect common processes in pancreatic NETs. Loss of SETD2 function and of trimethylation of H3K36 leads to defects in splicing and DNA repair due to failure of proteins that read this epigenetic modification to access chromatin [35]. Besides pancreatic NETs, *SETD2* mutations have been observed in other cancers, such as renal cell carcinomas, uterine carcinomas and others, arguing for the importance of the H3K36 methylation epigenetic mark for the suppression of carcinogenesis [38,39]. Therapeutically, in a synthetic lethality screen, cell lines with defective H3K36 methylation were sensitive to WEE1 kinase inhibitors due to induction of excessive replication stress [40]. WEE1 kinase regulates the CDK1/cyclin B complex to prevent entry into the mitosis phase of the cell cycle, and during the S phase, it regulates the CDK2 kinase to prevent replication stress. These data add tumors with *SETD2* mutations, including pancreatic NETs, to other synthetic lethal lesions that could be targeted by next-generation WEE1 kinase inhibitors with improved therapeutic windows [41]. WEE1 kinase inhibitors were synthetically lethal with defects in DNA damage response in pharmacologic screens and have advanced to clinical development in tumors with such defects. Hurdles in clinical development associated with excessive toxicity seem to have been partially overcome with the discovery of newer inhibitors displaying better adverse effect profiles [41]. These inhibitors remain in early-phase clinical development, and no data are available specifically for NETs.

A few samples with the highest mutation count in the MSK cohort showed significant contribution from the COSMIC SBS11 signature associated with temozolomide exposure. Temozolomide is a chemotherapeutic that is frequently used in NETs, either alone or in combination with capecitabine, and functions as a DNA methylator mostly in guanine nucleotides. Failure to correct the aberrant methylation by the cell’s repair systems results in cell apoptosis. If developed early after the start of treatment, the SBS11 signature could be a biomarker of efficacy of temozolomide in patients with pancreatic NETs, destined to have a good clinical response to the alkylating agent, and could complement the expression of O6-methylguanine methyltransferase (MGMT) or methylation of its promoter as a predictive biomarker of temozolomide therapy [42]. Notably, the increased mutation rate in the sub-set of temozolomide-exposed pancreatic NETs in the multi-institute cohort did not attain a level of hypermutability that would be expected to confer sensitivity to immune checkpoint inhibitors. In the multi-institute series, a recurring COSMIC SBS signature showing a high contribution in samples with the highest mutation burden was the BER-associated SBS36, despite the absence of *MUTYH* mutations in these cases. Some of the samples had mutations in *TP53* and *RB1*, supporting a component of dedifferentiation, as these mutations are frequently observed in poorly differentiated NETs. Germline *MUTYH* mutations and other germline mutations have been reported in neuroendocrine tumors [43]. Some patients with neuroendocrine tumors and germline mutations in *MUTYH* have higher TMB and display immune cell infiltration in the tumors. A response to immune checkpoint inhibitors has been noted in a few patients [43]. A multinational series of germline- and somatic *MUTYH*-mutated neuroendocrine tumor patients observed a low TMB, between zero and nine mutations/Mb, but also reported a significant increase in TMB following treatment with alkylating agents or protein radioligand therapy [44]. It would be interesting to test if this TMB increase is associated with immune checkpoint inhibitor sensitivity. 

The limitations of the study include the heterogeneity in the two cohorts analyzed and the genomic platforms used in their experiments. The multi-institute cohort used whole-exome sequencing and consisted mainly of localized tumors, whereas the MSK cohort used targeted sequencing and consisted largely of metastatic cases. Both cohorts were of moderate size, and some of the mutational subgroups of interest included only small numbers of patients, limiting the statistical power for discovering small or moderate differences. The mutational signature analysis was limited by the small number of mutations in each sample, especially in the MSK cohort that employed a targeted genomic panel; therefore, it should be interpreted with caution and be considered exploratory. In addition, the study described only a secondary computational analysis of published data, and no new data or wet experiments were contributed. Therefore, interesting questions that the source data were not designed to answer remain to be further studied.

In conclusion, the current report identifies genomic sub-sets of pancreatic NETs that could form the basis for targeted therapeutic interventions. Biomarkers of response to PI3K/mTOR pathway inhibitors, alkylating agents and targeted treatments for tumors with high CIN deserve further study. Therapies that would increase the TMB, which is very low at baseline in these tumors, followed by immune checkpoint inhibitor immunotherapy could be another therapeutic sequence to explore.

## 4. Materials and Methods

Two genomic series of pancreatic neuroendocrine tumors, including a multi-institute series from 3 countries (Italy, Australia and the United States of America), with 98 patients, and a series from the Memorial Sloan Kettering Cancer Center (MSKCC, MSK series), with 80 patients and a total of 96 samples (some patients had contributed more than one sample) formed the basis of the current analysis [45,46]. The multi-institute series employed whole-exome sequencing for genomic analyses, while the MSK series used a targeted sequence panel with 275 to 468 genes in different versions, called MSK-IMPACT (Memorial Sloan Kettering Integrated Mutation Profiling of Actionable Cancer Targets). Two variant calling algorithms (qSNP and GATK) were used for mutation identification in the multi-institute series [8]. Fraction Genome Altered (FGA), a metric for chromosomal instability in a sample, was defined as the fraction of the total sequenced genome length with copy number gains or losses. Data from the two series were accessed and analyzed in cBioportal, a cancer genomics portal (www.cbioportal.org, accessed on 7 June 2026) that hosts series of various cancers contributed by groups of investigators worldwide and made publicly available [47,48].

The single base substitution (SBS) contribution in individual samples was interrogated for identification of mutation signatures and fitted to the COSMIC (Catalogue of Somatic Mutations in Cancer, version v3.6) catalogue of signatures (https://cancer.sanger.ac.uk/signatures/, accessed on 7 June 2026) using established pipelines available in the R computational environment. The non-negative linear square method was used for signature fitting with the nnls R package. The COSMIC catalogue contains dozens of base substitution signatures derived from genomic studies of various types of cancers, with several signatures associated with causative exposures or defects in cellular DNA damage repair mechanisms [49]. The current version v3.6 contains 82 SBS signatures and 19 additional signatures that are considered potential artifacts.

Fisher’s exact test or the χ^2^ test was employed for statistical comparisons of categorical variables, and Student’s t test, the Kruskal–Wallis test or Analysis of Variance (ANOVA) was used for statistical comparisons of continuous variables. The Spearman correlation co-efficient was used for the statistical evaluation of correlations. The Bonferroni correction was applied to estimate the statistical significance of multiple comparisons. All statistical comparisons were considered significant when *p* was less than 0.05. 

## Figures and Tables

**Table 1:** Clinical and pathologic characteristics of the patients in the entire multi-institutional pancreatic NET cohort and in the groups with or without *MEN1* and *DAXX*/*ATRX* mutations. MA/D: group with both *MEN1* and *ATRX* or *DAXX* mutations, M: group with *MEN1* mutations without *ATRX* and *DAXX* mutations, A/D: group with *ATRX* or *DAXX* mutations without *MEN1* mutations, and None: group with no *MEN1*, *ATRX* or *DAXX* mutations. WHO: World Health Organization; NA: not available. The *p* values in the last column were obtained with the χ^2^ test, except if otherwise specified.

	Entire, *n* = 98 (%)	MA/D, *n* = 18 (%)	M, *n* = 18 (%)	A/D, *n* = 14 (%)	None, *n* = 48 (%)	*p*
Age						
Mean ± SD	57.3 ± 14.2	62.6 ± 11.7	62.3 ± 11	58.1 ± 13.5	53.2 ± 15.4	ANOVA *p* = 0.03
>65 years old	30 (30.6)	7 (38.9)	7 (38.9)	6 (42.9)	10 (20.8)	0.23
≤65 years old	68 (69.4)	11 (61.1)	11 (61.1)	8 (57.1)	38 (79.2)	
Sex						
Male	63 (64.3)	13 (72.2)	9 (50)	10 (71.4)	31 (64.6)	0.49
Female	35 (35.7)	5 (27.8)	9 (50)	4 (28.6)	17 (35.4)	
Stage						
I	42 (42.8)	5 (27.8)	11 (61.1)	4 (28.6)	22 (45.8)	0.12 (stage I–III versus IV)
II	37 (37.8)	7 (38.9)	7 (38.9)	5 (35.7)	18 (37.5)	
III	1 (1)	0	0	1 (7.1)	0	
IV	18 (18.4)	6 (33.3)	0	4 (28.6)	8 (16.7)	
WHO grade						
1	36 (36.7)	6 (33.3)	9 (50)	3 (21.4)	18 (37.5)	0.41 (Grade 1–2 versus grade 3)
2	57 (58.2)	12 (66.7)	9 (50)	11 (78.6)	25 (52.1)	
3	5 (5.1)	0	0	0	5 (10.4)	
Extra-pancreatic spread						
Present	45 (45.9)	11 (61.1)	5 (27.8)	10 (71.4)	19 (39.6)	0.03
Absent	53 (54.1)	7 (38.9)	13 (72.2)	4 (28.6)	29 (60.4)	
Vascular invasion						
Present	55 (59.8)	14 (87.5)	10 (55.6)	9 (69.2)	22 (48.9)	0.04
Absent	37 (40.2)	2 (12.5)	8 (44.4)	4 (30.8)	23 (51.1)	
NA	6	2		1	3	
Perineural invasion						
Present	36 (41.9)	8 (53.3)	4 (26.7)	7 (63.6)	17 (37.8)	0.19
Absent	50 (58.1)	7 (46.7)	11 (73.3)	4 (36.4)	28 (62.2)	
NA	12	3	3	3	3	

**Table 2:** Prevalence of frequent mutations in the entire multi-institutional pancreatic NET cohort and in the groups with or without *MEN1* and *DAXX*/*ATRX* mutations. MA/D: group with both *MEN1* and *ATRX* or *DAXX* mutations, M: group with *MEN1* mutations without *ATRX* and *DAXX* mutations, A/D: group with *ATRX* or *DAXX* mutations without *MEN1* mutations, and None: group with no *MEN1*, *ATRX* or *DAXX* mutations.

Gene	Entire, *n* = 98 (%)	MA/D, *n* = 18 (%)	M, *n* = 18 (%)	A/D, *n* = 14 (%)	None, *n* = 48 (%)
*MEN1*	36 (36.7)	18 (100)	18 (100)	0	0
*DAXX*	22 (22.4)	15 (83.3)	0	7 (50)	0
*ATRX*	10 (10.2)	3 (16.7)	0	7 (50)	0
*PTEN*	7 (7.1)	3 (16.7)	0	3 (21.4)	1 (2.1)
*SETD2*	5 (5.1)	0	2 (11.1)	2 (14.3)	1 (2.1)
*ATM*	3 (3.1)	2 (11.1)	0	0	1 (2.1)
*KMT2C*	3 (3.1)	2 (11.1)	0	0	1 (2.1)
*MED23*	3 (3.1)	2 (11.1)	0	1 (7.1)	0
*MTOR*	3 (3.1)	2 (11.1)	0	0	1 (2.1)
*NUMA1*	3 (3.1)	1 (5.6)	0	0	2 (4.2)
*RYR2*	3 (3.1)	0	1 (5.6)	2 (14.3)	0
*TP53*	3 (3.1)	1 (5.6)	0	0	2 (4.2)
*ARID1A*	2 (2)	0	0	1 (7.1)	1 (2.1)
*TP53BP1*	2 (2)	0	0	2 (14.3)	0
*TSC2*	2 (2)	0	0	1 (7.1)	1 (2.1)
*AMOTL2*	2 (2)	1 (5.6)	1 (5.6)	0	0
*BCL2L14*	2 (2)	0	0	1 (7.1)	1 (2.1)
*BCOR*	2 (2)	0	1 (5.6)	1 (7.1)	0
*CDKN1A*	2 (2)	0	1 (5.6)	1 (7.1)	0
*DDR2*	2 (2)	2 (11.1)	0	0	0
*FAT4*	2 (2)	0	0	1 (7.1)	1 (2.1)
*KAT6A*	2 (2)	0	0	0	2 (4.2)
*RB1*	2 (2)	0	0	0	2 (4.2)
*SMARCB1*	2 (2)	1 (5.6)	0	0	1 (2.1)
*TSC1*	2 (2)	0	0	1 (7.1)	1 (2.1)

**Table 3:** Dominant COSMIC SBS signatures in the multi-institute cohort. SNVs: single nucleotide variants, BER: base excision repair, HRD: homologous recombination deficiency, TMB: tumor mutation burden, and MMR: mismatch repair.

Signature(s)	Etiology	Total SNVs	% Samples	Comments
SBS36	BER deficiency (*MUTYH*)	344 (16%)	12.4%	Dominant in samples with high TMB
SBS3	HRD/BRCA deficiency	245 (12%)	41.2%	Broad, low-level contribution across cohort
SBS1	Clock/deamination	225 (11%)	52.6%	Ubiquitous aging signal
SBS5	Clock/aging	161 (8%)	32%	Ubiquitous aging signal
SBS31	Platinum chemotherapy	141 (7%)	33%	Treatment-related
SBS15/SBS6/SBS26	MMR deficiency	319 (15%) combined	23–34% each	MMR-deficient signatures present low-level contributions in a fourth to a third of samples
SBS40a/b/c	Aging	205 (10%) combined	10–21% each	Background aging
SBS13/SBS2	APOBEC	140 (7%) combined	26–32%	Low level in multiple samples
SBS18	Oxidative stress/ BER deficiency	70 (3%)	19.6%	Related to SBS36

**Table 4:** The 9 samples with the highest tumor mutation burden (TMB) in the multi-institute series. Genes or gene families that are recurrently mutated are presented in bold in the last column.

Sample ID	Dominant Substitution	% Dominant Substitution	TMB (Mutations/Mb)	MEN1/ATRX/DAXX	Other Cancer-Associated Mutated Genes
ITNET_0124	C>A	84 (68 of 81)	2.83	MD	*SMAD4*, *ATM*, *FOXO1*, *GRB7*, ***KMT2C***, *RICTOR*
ITNET_0052	C>A	81.8 (108 of 132)	4.5	MA	***TP53***, ***KMT2C***, *ERBB2*, *INHBA*, *SOX9*, *STAT6*, ***CDH17***, *GLI2*
NE_0033	C>A	81.8 (72 of 88)	3.03	D	*CDKN1A*, ***PTEN***, *SETD2*, *BCOR*, *PIK3C3*, *TP53BP1*, *RYR2*
ICGC_0452	C>A	85.7 (54 of 63)	2.1	D	*ATR*, *HNF1A*, *BRIP1*, *BMP3*, *BMPR1B*, *NFATC1*
IT_0938	C>T	83.3 (100 of 120)	4.1	M	*CDKN1A*, *NOTCH1*, *NOTCH3*, *GATA6*, *MSH6*, ***SMARCA2***, *MTHFR*, *KIF1A*, ***CDH1***
ICGC_0455	C>T	42.9 (36 of 84)	3.03	None	***TP53***, ***ARID2***, ***RB1***, *TOP1*, *KMT2C*, *ITGB6*, *SMAD5*, *SALL1*, *SOX11*
ITNET_0100	C>T	31.8 (28 of 88)	2.7	None	***CDH6***, ***CDH16***, *DACH1*, *IL2RG*, *ZEB1*
ITNET_1047	C>T	31.3 (21 of 67)	2.3	None	***TP53***, ***RB1***, *MAD2L2*, *MET*, *PARP4*, *RUNX3*
ITNET_1308	C>T	53.8 (28 of 52)	1.9	MD	***PTEN***, ***SMARCB1***, *EP400*, *BIRC5*, *ZNF292*

**Table 5:** Clinical and pathologic characteristics of the patients in the entire MSK pancreatic NET cohort and in the groups with or without *MEN1* and *DAXX*/*ATRX* mutations. MA/D: group with both *MEN1* and *ATRX* or *DAXX* mutations, M: group with *MEN1* mutations without *ATRX* and *DAXX* mutations, A/D: group with *ATRX* or *DAXX* mutations without *MEN1* mutations, and None: group with no *MEN1*, *ATRX* or *DAXX* mutations. The *p* values in the last column were obtained with the χ^2^ test, except if otherwise specified.

	Entire, *n* = 80 (%)	MA/D, *n* = 36 (%)	M, *n* = 8 (%)	A/D, *n* = 11 (%)	None, *n* = 25 (%)	*p*
Age						
Median (IQR)	53.5 (43–63)	55 (47–68)	63 (58.5–66.2)	61 (44.5–64.5)	47 (33–53)	ANOVA < 0.001
>65 yo	16 (20)	11 (30.6)	2 (25)	3 (27.3)	0	0.07
≤65 yo	64 (80)	25 (69.4)	6 (75)	8 (62.7)	25 (100)	
Sex						
Male	35 (43.7)	16 (44.4)	4 (50)	8 (72.7)	7 (28)	
Female	45 (56.3)	20 (55.6)	4 (50)	3 (27.3)	18 (72)	0.1
WHO grade						
1	27 (33.8)	12 (33.3)	5 (62.5)	5 (45.5)	5 (20)	0.09 (Grade 1–2 versus grade 3)
2	40 (50)	20 (55.6)	2 (25)	5 (45.5)	13 (52)	
3	13 (16.2)	4 (11.1)	1 (12.5)	1 (9)	7 (28)	
Primary tumor location						
Head	19 (23.7)	7 (19.4)	0	2 (18.2)	10 (40)	0.09
Body/tail/uncinate	61 (76.3)	29 (80.6)	8 (100)	9 (81.8)	15 (60)	
Liver metastases						
Present	66 (82.5)	32 (88.9)	7 (87.5)	9 (81.8)	18 (72)	0.4
Absent	14 (17.5)	4 (11.1)	1 (12.5)	2 (18.2)	7 (28)	
Ki-67 index (%), median (IQR)	10 (3–21.6)	15 (4–20)	3 (2–9)	14.4 (4.8–19.6)	10 (5–32.5)	Kruskal–Wallis 0.36
Mutation count, median (IQR)	3 (2–5)	4 (3–6)	1 (1–5)	3 (1.5–3.8)	2 (1–3.5)	Kruskal–Wallis 0.016
Fraction Genome Altered, median (IQR)	0.6 (0.2–0.9)	0.8 (0.7–0.9)	0.7 (0.6–0.9)	0.6 (0.3–0.8)	0.2 (0.1–0.3)	Kruskal–Wallis <0.001

**Table 6:** Prevalence of frequent mutations in the entire MSK pancreatic NET cohort and in the groups with FGA below 0.35 (31 patients) and above 0.6 (44 patients). Five patients with FGA between 0.35 and 0.6 were excluded from the analysis. In the last column, the q value, corrected for multiple comparisons, is given in parentheses for the comparisons where *p* is significant.

Gene	Entire, *n* = 80 (%)	FGA < 0.35, *n* = 31 (%)	FGA > 0.6, *n* = 44 (%)	*p* (q)
*MEN1*	41 (51.3)	6 (19.4)	33 (75)	<0.0001 (0.002)
*DAXX*	32 (40)	6 (19.4)	24 (54.5)	0.003 (0.07)
*ATRX*	20 (25)	5 (16.1)	15 (34.1)	0.11
*PTEN*	9 (11.3)	1 (3.2)	9 (20.5)	0.03 (0.7)
*SETD2*	14 (17.5)	9 (29)	4 (9.1)	0.03 (0.7)
*ATM*	6 (7.5)	2 (6.5)	3 (6.8)	1
*KMT2C*	7 (8.7)	1 (3.2)	6 (13.6)	0.22
*MTOR*	3 (3.8)	0	3 (6.8)	0.26
*TP53*	10 (12.5)	1 (3.2)	7 (15.9)	0.12
*ARID1A*	9 (11.3)	4 (12.9)	5 (11.4)	1
*TSC2*	17 (21.3)	1 (3.2)	16 (36.4)	0.0006 (0.01)
*RB1*	1 (1.2)	0	1 (2.3)	1
*TSC1*	4 (5)	2 (6.5)	2 (4.5)	1
*BRAF*	6 (7.5)	4 (12.9)	3 (6.8)	0.43
*KMT2D*	6 (7.5)	3 (9.7)	2 (4.5)	0.64
*MED12*	6 (7.5)	2 (6.5)	4 (9.1)	1
*ARID2*	5 (6.3)	0	5 (11.4)	0.07
*BRCA2*	5 (6.3)	1 (3.2)	4 (9.1)	0.39
*NOTCH1*	5 (6.3)	2 (6.5)	3 (6.8)	1
*NOTCH3*	5 (6.3)	3 (9.7)	2 (4.5)	0.64
*NOTCH4*	4 (5)	1 (3.2)	2 (4.5)	1
*TERT*	5 (6.3)	2 (6.5)	4 (9.1)	1
*CREBBP*	4 (5)	0	4 (9.1)	0.13
*CBL*	4 (5)	1 (3.2)	2 (4.5)	1
*ERBB4*	4 (5)	1 (3.2)	2 (4.5)	1
*FAT1*	4 (5)	3 (9.7)	1 (2.3)	0.3
*IGF1R*	4 (5)	1 (3.2)	2 (4.5)	1
*KRAS*	4 (5)	0	3 (6.8)	0.26
*LATS2*	4 (5)	2 (6.5)	2 (4.5)	1
*YAP1*	4 (5)	2 (6.5)	3 (6.8)	1
*VHL*	4 (5)	2 (6.5)	2 (4.5)	1
*SMAD4*	4 (5)	0	3 (6.8)	0.26

**Table 7:** Prevalence of frequent copy number alterations in the entire MSK pancreatic NET cohort and in the groups with FGA below 0.35 (31 patients) and above 0.6 (44 patients). Five patients with FGA between 0.35 and 0.6 were excluded from the analysis.

Gene	Entire, *n* = 80 (%)	FGA < 0.35, *n* = 31 (%)	FGA > 0.6, *n* = 44 (%)	*p*
Deletions				
*MEN1*	3 (3.8)	0	3 (6.8)	0.26
*CDKN2A*	13 (16.3)	4 (12.9)	11 (25)	0.24
*CDKN2B*	14 (17.5)	4 (12.9)	12 (27.3)	0.16
*IRF4*	4 (5)	2 (6.5)	3 (6.8)	1
*TSC2*	2 (2.4)	1 (3.2)	2 (4.5)	1
Amplifications				
*GNAS*	3 (3.8)	0	3 (6.8)	0.26
*PLK2*	3 (3.8)	0	3 (6.8)	0.26

**Table 8:** Treatments and outcomes of the patients in the entire MSK pancreatic NET cohort and in the groups with or without *MEN1* and *DAXX*/*ATRX* mutations. MA/D: group with both *MEN1* and *ATRX* or *DAXX* mutations, M: group with *MEN1* mutations without *ATRX* and *DAXX* mutations, A/D: group with *ATRX* or *DAXX* mutations without *MEN1* mutations, and None: group with no *MEN1*, *ATRX* or *DAXX* mutations. The *p* values in the last column were obtained with the χ^2^ test, except if otherwise specified.

	Entire, *n* = 80 (%)	MA/D, *n* = 36 (%)	M, *n* = 8 (%)	A/D, *n* = 11 (%)	None, *n* = 25 (%)	*p*
Previous surgery						
Yes	48 (60)	21 (58.3)	4 (50)	8 (72.7)	15 (60)	0.8
No	32 (40)	15 (41.7)	4 (50)	3 (27.3)	10(40)	
Somtostatin analog						
Yes	58 (72.5)	29 (80.6)	8 (100)	6 (54.5)	15 (60)	0.04
No	22 (27.5)	7 (19.4)	0	5 (45.5)	10 (40)	
Hepatic artery embolization						
Yes	35 (43.8)	16 (44.4)	3 (37.5)	4 (36.4)	12 (48)	0.9
No	45 (56.2)	20 (55.6)	5 (62.5)	7 (63.6)	13 (52)	
Targeted therapy						
Yes	31 (38.8)	16 (44.4)	2 (25)	4 (36.4)	9 (36)	0.77
No	49 (61.2)	20 (55.6)	6 (75)	7 (63.6)	16 (64)	
PRRT						
Yes	12 (15.2)	5 (14.3)	0	1 (9.1)	6 (24)	0.4
No	67 (84.8)	30 (85.7)	8 (100)	10 (90.9)	19 (76)	
NA	1	1				
Chemotherapy						
Yes	48 (60.8)	22 (62.9)	2 (25)	7 (63.6)	17 (68)	0.19
No	31 (39.2)	13 (37.1)	6 (75)	4 (36.4)	8 (32)	
NA	1	1				
Survival status						
Living	66 (82.5)	32 (88.9)	6 (75)	10 (90.9)	18 (72)	0.27
Deceased	14 (17.5)	4 (11.1)	2 (25)	1 (9.1)	7 (28)	
Median OS in months (IQR)	39.2 (18.8–80)	73.9 (26.8–89.7)	36.3 (29.3–60.7)	40.5 (16.3–86.3)	28.6 (15.1–40.6)	Kruskal–Wallis 0.014

**Table 9:** Prevalence of frequent mutations in the entire MSK pancreatic NET cohort and in the groups with or without *MEN1* and *DAXX*/*ATRX* mutations. MA/D: group with both *MEN1* and *ATRX* or *DAXX* mutations, M: group with *MEN1* mutations without *ATRX* and *DAXX* mutations, A/D: group with *ATRX* or *DAXX* mutations without *MEN1* mutations, and None: group with no *MEN1*, *ATRX* or *DAXX* mutations. In the last column, the q value, corrected for multiple comparisons, is given in parentheses for the comparisons where *p* is significant.

Gene	Entire, *n* = 80 (%)	MA/D, *n* = 36 (%)	M, *n* = 8 (%)	A/D, *n* = 11 (%)	None, *n* = 25 (%)	*p* (q)
*MEN1*	41 (51.3)	35 (97.2)	6 (75)	0	0	<0.001 (0.02)
*DAXX*	32 (40)	25 (69.4)	0	7 (63.6)	0	<0.001 (0.02)
*ATRX*	20 (25)	15 (41.7)	0	5 (45.5)	0	<0.001 (0.02)
*PTEN*	9 (11.3)	7 (19.4)	1 (12.5)	1 (9.1)	0	0.08
*SETD2*	14 (17.5)	8 (22.2)	0	2 (18.2)	4 (16)	0.63
*ATM*	6 (7.5)	3 (8.3)	0	1 (9.1)	2 (8)	1
*KMT2C*	7 (8.7)	4 (11.1)	1 (12.5)	2 (18.2)	0	0.15
*MTOR*	3 (3.8)	3 (8.3)	0	0	0	0.45
*TP53*	10 (12.5)	7 (19.4)	0	2 (18.2)	1 (4)	0.21
*ARID1A*	9 (11.3)	5 (13.9)	0	2 (18.2)	2 (8)	0.64
*TSC2*	17 (21.3)	13 (36.1)	0	3 (27.3)	1 (4)	0.006 (0.09)
*RB1*	1 (1.2)	1 (2.8)	0	0	0	1
*TSC1*	4 (5)	2 (5.6)	0	1 (9.1)	1 (4)	0.77
*BRAF*	6 (7.5)	2 (5.6)	0	1 (9.1)	3 (12)	0.65
*KMT2D*	6 (7.5)	2 (5.6)	1 (12.5)	1 (9.1)	2 (8)	0.81
*MED12*	6 (7.5)	4 (11.1)	0	1 (9.1)	1 (4)	0.69
*ARID2*	5 (6.3)	3 (8.3)	1 (12.5)	1 (9.1)	0	0.27
*BRCA2*	5 (6.3)	3 (8.3)	0	2 (18.2)	0	0.15
*NOTCH1*	5 (6.3)	3 (8.3)	0	1 (9.1)	1 (4)	0.82
*NOTCH3*	5 (6.3)	2 (5.6)	0	2 (18.2)	1 (4)	0.36
*NOTCH4*	4 (5)	3 (8.3)	0	0	1 (4)	0.88
*TERT*	5 (6.3)	2 (5.6)	0	2 (18.2)	1 (4)	0.36
*CREBBP*	4 (5)	4 (11.1)	0	0	0	0.28
*CBL*	4 (5)	3 (8.3)	0	1 (9.1)	0	0.35
*ERBB4*	4 (5)	2 (5.6)	0	1 (9.1)	1 (4)	0.77
*FAT1*	4 (5)	0	1 (12.5)	1 (9.1)	2 (8)	0.13
*IGF1R*	4 (5)	1 (2.8)	1 (12.5)	0	2 (8)	0.51
*KRAS*	4 (5)	3 (8.3)	0	0	1 (4)	0.88
*LATS2*	4 (5)	2 (5.6)	0	1 (9.1)	1 (4)	0.77
*YAP1*	4 (5)	2 (5.6)	0	1 (9.1)	1 (4)	0.76
*VHL*	4 (5)	1 (2.8)	1 (12.5)	0	2 (8)	0.51
*SMAD4*	4 (5)	3 (8.3)	0	1 (9.1)	0	0.35

**Table 10:** Prevalence of frequent copy number alterations in the entire MSK pancreatic NET cohort and in the groups with or without *MEN1* and *DAXX*/*ATRX* mutations. MA/D: group with both *MEN1* and *ATRX* or *DAXX* mutations, M: group with *MEN1* mutations without *ATRX* and *DAXX* mutations, A/D: group with *ATRX* or *DAXX* mutations without *MEN1* mutations, and None: group with no *MEN1*, *ATRX* or *DAXX* mutations.

Gene	Entire, *n* = 80 (%)	MA/D, *n* = 36 (%)	M, *n* = 8 (%)	A/D, *n* = 11 (%)	None, *n* = 25 (%)	*p*
Deletions						
*MEN1*	3 (3.8)	1 (2.8)	2 (25)	0	0	0.03
*CDKN2A*	13 (16.3)	6 (16.7)	0	4 (36.4)	3 (12)	0.18
*CDKN2B*	14 (17.5)	7 (19.4)	0	4 (36.4)	3 (12)	0.2
*IRF4*	4 (5)	4 (11.1)	0	0	0	0.29
*TSC2*	2 (2.4)	1 (2.8)	1 (12.5)	0	0	0.29
Amplifications						
*GNAS*	3 (3.8)	3 (8.3)	0	0	0	0.39
*PLK2*	3 (3.8)	3 (8.3)	0	0	0	0.39

## Data Availability

The original contributions presented in this study are included in this article. Further inquiries can be directed to the corresponding author.
